# A Case of Severe Neonatal COVID-19 Pneumonia Requiring Prolonged Mechanical Ventilation Without Long-Term Respiratory Sequelae

**DOI:** 10.7759/cureus.99444

**Published:** 2025-12-17

**Authors:** Tomoko Okada, Sho Kimura, Takafumi Honda, Kumi Yasukawa, Jun-Ichi Takanashi

**Affiliations:** 1 Department of Pediatrics, Tokyo Women's Medical University Yachiyo Medical Center, Yachiyo, JPN

**Keywords:** covid-19, mechanical ventilation, neonatal, pneumonia, sequelae, serum klebs von den lungen-6 (kl-6)

## Abstract

Numerous reports have described severe effects of novel coronavirus (SARS-CoV-2) in adults, often accompanied by long-term respiratory sequelae, such as pulmonary fibrosis. By stark contrast, most pediatric cases of respiratory illness associated with SARS-CoV-2 are mild: severe pneumonia caused by SARS-CoV-2 infection is rare in children. Nevertheless, data reflecting long-term respiratory outcomes in this population are scarce. This report describes prolonged mechanical ventilation for managing a case of severe neonatal pneumonia caused by SARS-CoV-2. The patient, a full-term neonate who developed fever at two days of age, was subsequently diagnosed with moderate acute respiratory distress syndrome (ARDS) secondary to COVID-19. Mechanical ventilation was administered for three weeks. Following extubation, the patient’s respiratory condition improved steadily. He was discharged without apparent sequelae. At two-year follow-up, he had no respiratory or other systemic sequelae. To monitor for potential long-term pulmonary damage, we performed serial chest computed tomography (CT) scans and measured serum KL-6 levels. The latter were elevated during the acute phase (2,600 U/mL) but decreased to 450 U/mL within three months. CT imaging initially showed ground-glass opacities, which resolved over time. This case highlights the possibility of full recovery without long-term respiratory complications, even in neonates with severe SARS-CoV-2 pneumonia requiring prolonged ventilatory support. This report is among the very few documenting long-term follow-up in this patient population.

## Introduction

Children generally experience milder symptoms of coronavirus disease 2019 (COVID-19) than adults [1], with most cases ranging from asymptomatic to mild illness, subsequently resolving within one to two weeks. However, as the pandemic progressed and as pediatric cases increased, reports of severe disease with severe complications and long-term effects in children emerged [2,3]. Although cases of so-called “long COVID” in children have been reported, data related to the long-term prognosis and respiratory outcomes in severe pediatric cases are lacking [4].

Neonatal cases of COVID-19 remain rare. The reported hospitalization rate for neonates with SARS-CoV-2 infection is approximately 5.6 per 10,000 live births [5]. A report of a large CDC study described that during Delta predominance (July-November 2021), the average weekly age-standardized incidence of COVID-19 cases was 460.1 per 100,000 unvaccinated adults [6].

Most affected neonates exhibit only mild symptoms such as gastrointestinal disturbances or feeding difficulties [5]. Pneumonia requiring mechanical ventilation in this population is exceedingly uncommon. Earlier reports of neonatal cases describe the typical duration of mechanical ventilation as approximately one week [7]. Additionally, clinical outcomes are comparable to those of pneumonia caused by other respiratory viruses. Published evidence remains limited to case reports and small case series. Although several recent pediatric COVID-19 management guidelines and consensus statements have been published, recommendations specific to the neonatal period remain limited [8]. Evidence supporting ventilatory strategies in this age group is still inadequate.

Growing evidence related to long-term pulmonary sequelae of COVID-19 in adults has been accumulated in recent years, particularly for patients who develop acute respiratory distress syndrome (ARDS). Pulmonary fibrosis has been identified as a potential long-term complication. Elevated serum levels of KL-6, a biomarker of interstitial lung disease, have been associated with persistent abnormal findings of chest computed tomography (CT) scans taken 12 weeks after infection. These findings suggest that serum KL-6 might serve as a useful marker for evaluating pulmonary sequelae without the need for frequent imaging [9].

By contrast, reports of severe SARS-CoV-2 pneumonia in children remain limited. Data related to long-term respiratory outcomes in this population are scarce. While multisystem inflammatory syndrome in children (MIS-C) and long COVID have been well documented [3], few studies have addressed the potential for lasting pulmonary damage in pediatric patients who receive mechanical ventilation.

This report describes a rare case of severe neonatal SARS-CoV-2 pneumonia requiring prolonged mechanical ventilation during the Delta variant wave of COVID-19. Despite the need for extended respiratory support, no residual respiratory abnormality was observed, as inferred from follow-up chest CT imaging and serum KL-6 levels. However, no earlier report of the relevant literature has detailed a study of longitudinal radiological and biomarker-based assessments in neonates recovering from severe COVID-19 pneumonia.

## Case presentation

For this male neonate born at 38 weeks and four days of gestation via vaginal delivery, the perinatal course was uneventful: the infant roomed in with the mother following birth. On day two of life, the infant developed a fever. Blood, sputum, urine, and cerebrospinal fluid cultures were all negative. The fever had resolved by day three. However, his peripheral oxygen saturation (SpO_2_) was persistently approximately 90%, prompting initiation of oxygen therapy.

On day five of life, reverse transcription polymerase chain reaction (RT-PCR) confirmed SARS-CoV-2 infection. Chest radiography revealed findings consistent with pneumonia. By day seven, his SpO_2_ had declined. Nasal continuous-positive airway pressure (nCPAP) was initiated along with intravenous remdesivir. The initial dose of remdesivir was set as 2.5 mg/kg/day [10]. On day eight, he was unable to maintain SpO_2_ above 90% with FiO_2_ of 0.5, necessitating endotracheal intubation and transfer to the pediatric intensive care unit (PICU).

**Figure 1:**
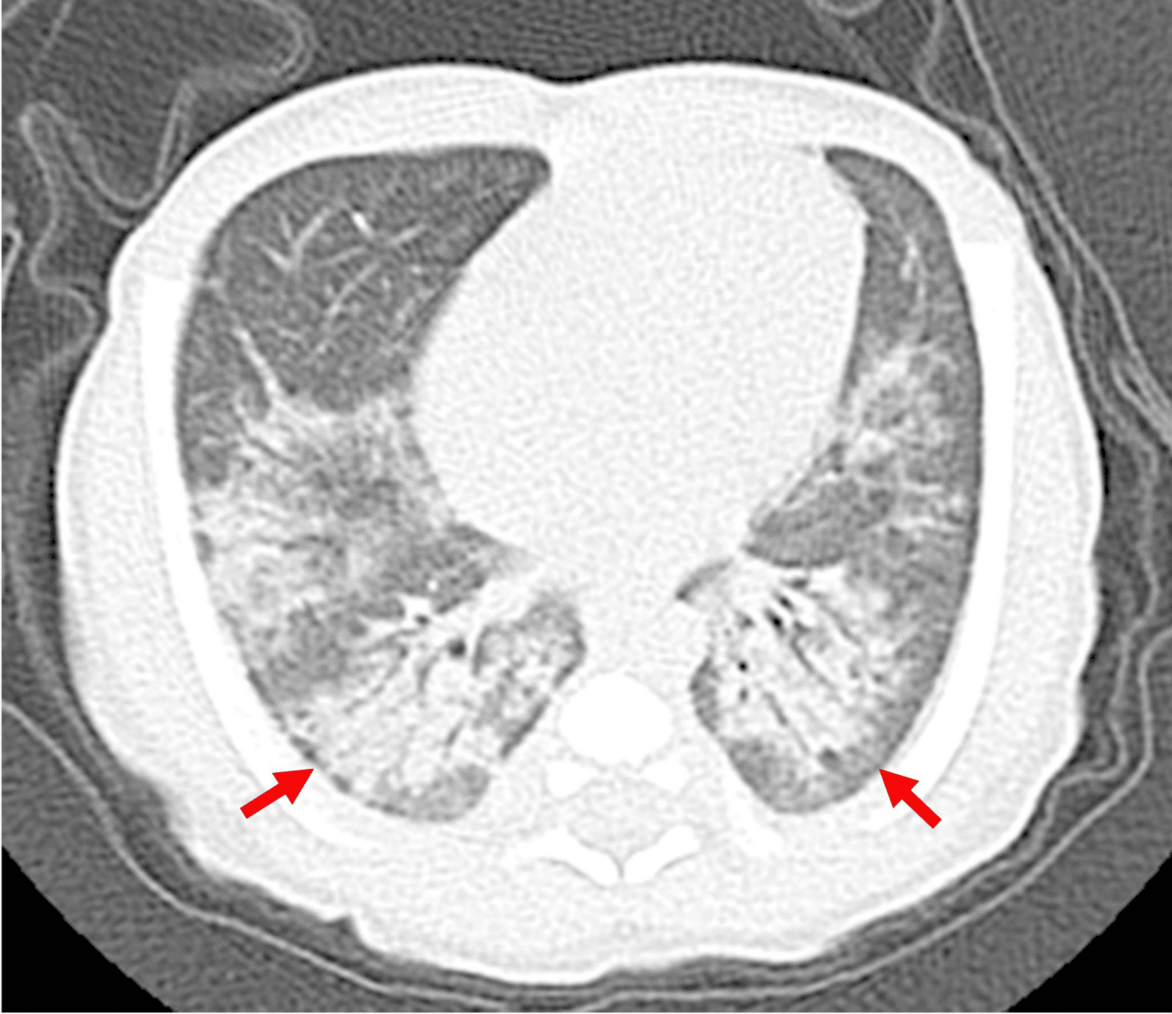
Chest computed tomography from day five Infiltrative shadows with air bronchograms are visible throughout the dorsal lung fields, suggesting pneumonia in these regions. The arrow indicates an area in which the subpleural area is spared from the infiltrative shadow. Ground-glass opacities are present in the surrounding tissue. Bronchial wall thickening was also observed.

Chest computed tomography (CT) revealed bilateral perihilar and dorsal lung consolidations (Figure 1). On the same day, the patient continued to receive remdesivir (1.25 mg/kg/day) and began receiving dexamethasone (0.3 mg/kg/day), heparin (10 U/kg/h), and cefotaxime. Based on findings of dorsal atelectasis, prone positioning was implemented for 16 hours per day.

**Figure 2:**
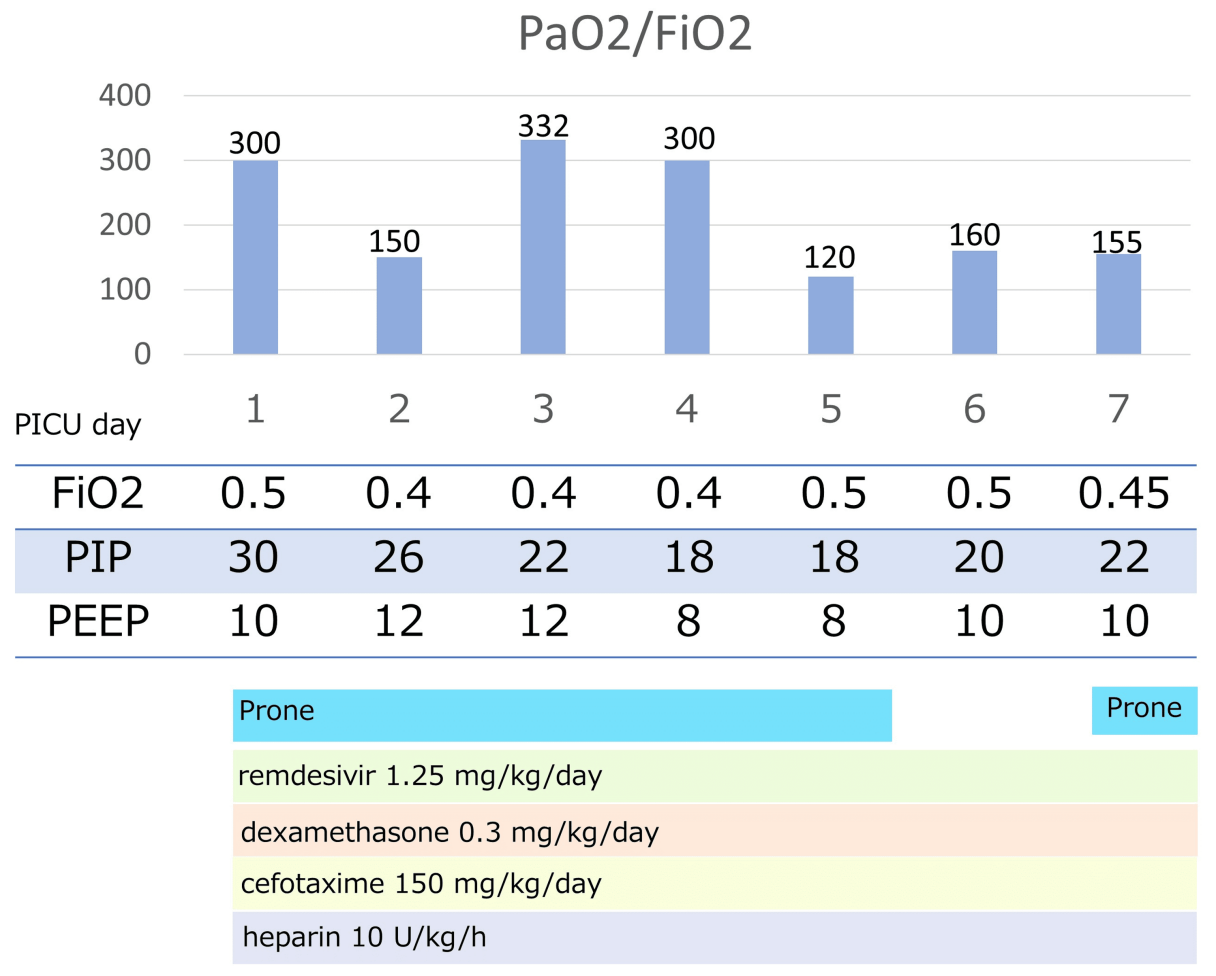
Clinical course after admission to the PICU Respiratory settings and treatment details from PICU admission to the extracorporeal membrane oxygenation (ECMO) center are shown.

Upon PICU admission, the patient was managed with peak inspiratory pressure (PIP) of 30 cmH_2_O, positive end-expiratory pressure (PEEP) of 10 cmH^2^O, and FiO_2_ of 0.5, with maintained PaCO_2_ of 40-50 mmHg. The oxygenation index (OI) was 15. The PaO_2_ /FiO_2_ ratio was 100-200. Prone positioning improved SpO_2_ from 93-95% to >96%, with a corresponding increase in PaO_2_ from 80 to 100 mmHg and a reduction in OI from 15 to 5, indicating clinical benefit. However, on day five of PICU admission, his respiratory status deteriorated after returning to the supine position: OI rose to 8. Because of worsening ventilation despite standard management, transfer to an extracorporeal membrane oxygenation (ECMO) center was considered. Neuromuscular blockade was initiated. Figure 2 shows the ventilator settings and treatment details after admission to the PICU.

The patient's condition improved gradually without the need for ECMO. He was extubated on day 29 of life and was transitioned to a high-flow nasal cannula (HFNC), which was discontinued on day 55. The patient was discharged home on day 61 without requiring supplemental oxygen.

This case occurred during the SARS-CoV-2 Delta variant epidemic. No member of his family had received the COVID-19 vaccination. Genomic sequencing of the virus was not performed. To investigate the route of infection, SARS-CoV-2 IgM and IgG antibodies (Abbott ARCHITECT assay) were measured on days 2 and 40. The patient tested positive for SARS-CoV-2 by RT-PCR. The mother also tested positive shortly thereafter. Among other household contacts, only the older brother tested positive. The negative IgM result excluded in-utero transmission, but vertical transmission during vaginal delivery or horizontal transmission after birth could not be ruled out.

**Figure 3:**
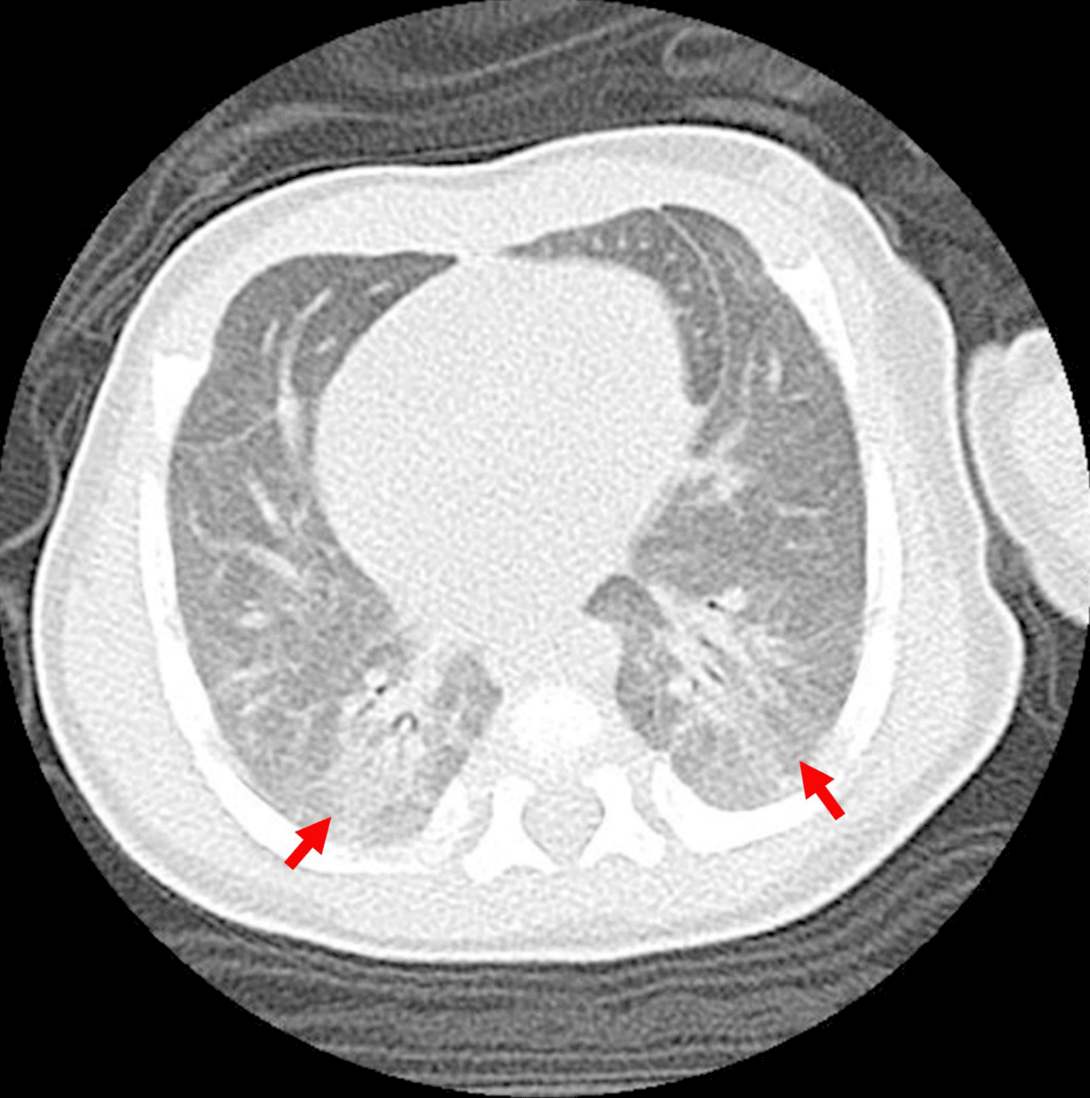
Chest computed tomography performed one year after discharge The arrows indicate that the schematic infiltrative changes were only visible in the bilateral lower lobes.

To assess long-term respiratory sequelae, we monitored serum KL-6 levels and conducted follow-up chest CT imaging. Although KL-6 concentrations were markedly elevated during the acute phase (2,600 U/mL; neonatal reference: 50.8-226.3 U/mL), they had decreased to 560 U/mL at discharge (infant reference: 83.7-249.9 U/mL). Those concentrations continued to decline, reaching 450 U/mL at three months and 360 U/mL at one year after onset. A chest CT scan performed one year after disease onset showed only minor residual infiltrative changes in the bilateral lower lobes (Figure 3).

One year after discharge, the patient contracted human metapneumovirus but experienced only mild respiratory symptoms without hospitalization. Neurological and motor development have remained age-appropriate throughout a two-year follow-up period.

## Discussion

This report describes a case of severe neonatal COVID-19 pneumonia requiring prolonged mechanical ventilation for three weeks, with no apparent long-term respiratory sequela. In neonates, respiratory sequelae are not limited to fibrosis but might include bronchopulmonary dysplasia, recurrent wheezing, or delayed alveolar development secondary to prolonged mechanical ventilation or nutritional compromise. No such complication was observed in this case. No specific post-discharge intervention was necessary.

Compared to neonatal cases reported earlier during the Delta variant epidemic, this case presented with more severe hypoxemia and required longer respiratory support. To date, no established guideline exists for mechanical ventilation management in neonates with COVID-19 pneumonia. Published evidence remains limited to case reports and small case series [11]. Therefore, management in this case was guided by general pediatric SARS-CoV-2 treatment protocols. Although loop-mediated isothermal amplification (LAMP) assays have been reported as a useful diagnostic tool for SARS-CoV-2 [12], particularly in pediatric settings, the infection in this case was confirmed using RT-PCR, providing high diagnostic specificity.

Given the presence of dorsal atelectasis observed on chest CT imaging, prone positioning was initiated. Oxygenation markedly improved following the positional change, suggesting a beneficial effect. Prone positioning, an established intervention for patients with moderate-to-severe ARDS, has been associated with improved oxygenation and reduced mortality when applied early and for prolonged sessions in severe ARDS (PROSEVA trial) [13]. More recent clinical studies indicate that prone positioning produces comparable physiological benefits in patients with COVID-19-related ARDS, including rapid and clinically meaningful improvements in PaO₂/FiO₂ and static respiratory system compliance. Accordingly, given the presence of dorsal atelectasis on chest CT in our patient, prone positioning was adopted and was followed by immediate improvement in oxygenation in this neonate [14].

Some reports have described similar benefits for neonates [7]. Knowledge obtained from treating this case adds to the emerging evidence supporting prone positioning as a useful adjunctive therapy in neonates with SARS-CoV-2 pneumonia. Further investigation into its efficacy in this population is warranted.

Because the clinical course was more severe than typically reported for neonates, we conducted long-term follow-up to assess possible respiratory sequelae, including serial chest CT imaging and measurement of serum KL-6 levels. In adults with COVID-19, ground-glass opacities (GGO) and pulmonary fibrosis have been reported as residual findings from CT scans performed 12 weeks after symptom onset [9]. However, in this case, follow-up imaging during the subacute phase was deferred to minimize radiation exposure. A chest CT performed one year after disease onset showed only subtle, non-specific infiltrative changes without evidence of GGO or fibrosis. These findings suggest that long-term pulmonary outcomes in neonates might differ from those observed in adults, even in severe cases.

Serum KL-6, a biomarker of interstitial lung disease, was also used to monitor disease severity and recovery. In adult patients with COVID-19, serum KL-6 levels are elevated considerably in severe cases compared to moderate cases, with a proposed cutoff of 335 U/mL for predicting severity [15]. Furthermore, persistent elevation of KL-6 during convalescence has been associated with radiographic sequelae. No pediatric-specific cutoff values exist, but the KL-6 level found in our patient peaked at 2,600 U/mL during the acute phase, markedly exceeding reference values for neonates (50.8-226.3 U/mL) and infants (83.7-249.9 U/mL). This elevation was consistent with the illness severity and the prolonged duration of mechanical ventilation. Subsequent normalization of KL-6 levels over time occurred parallel to the clinical recovery and with the absence of radiographic sequelae, supporting its potential role as a biomarker indicating both severity and prognosis.

Importantly, pulmonary function testing could not be performed because of the patient’s age and inability to follow instructions. Nonetheless, the child remained asymptomatic, demonstrated normal growth and development, and exhibited no recurrence of lower respiratory tract infections during a two-year follow-up period. These findings led to the clinical determination that no functional respiratory sequela had occurred.

There remains a significant gap in the literature regarding post-intensive care syndrome (PICS) among pediatric patients with COVID-19 pneumonia requiring endotracheal intubation. While adult studies have reported on the occurrence and risk factors of PICS, clinical characteristics, and risk factors of PICS [16], evidence in children is largely restricted to acute-phase outcomes. Minimal data address long-term neurocognitive, physical, and psychosocial sequelae. Few investigations have examined persistent functional impairment following pediatric critical illness related to SARS-CoV-2 infection. Therefore, longitudinal studies focusing specifically on the pediatric population are necessary to clarify the trajectory of post-ICU morbidity and inform strategies for the timely identification, prevention, and rehabilitation of PICS in this vulnerable group.

This case underscores several key considerations in the management and follow-up of severe neonatal COVID-19 pneumonia. Particularly, serum KL-6 might serve as a valuable, noninvasive biomarker for assessing both disease severity in the acute phase and potential sequelae during recovery, especially in populations for whom modalities of imaging or pulmonary function testing are limited. As the pandemic continues to evolve, further accumulation and analysis of similar cases will be crucially important for informing evidence-based treatment strategies and for establishing guidelines for managing severe neonatal SARS-CoV-2 pneumonia.

## Conclusions

This report describes a rare case of neonatal pneumonia caused by SARS-CoV-2 infection requiring prolonged mechanical ventilation for three weeks. Despite the extended course, the patient showed complete clinical and radiological recovery, with no evidence of long-term respiratory sequelae. Serial measurement of serum KL-6 levels correlated with disease severity and recovery, suggesting its potential utility as a noninvasive biomarker for monitoring pulmonary involvement and for minimizing the need for repeated imaging in pediatric patients. Further studies must be conducted to validate KL-6 as a prognostic tool for neonatal COVID-19 pneumonia.
